# Carbon and Nitrogen Mineralization in Relation to Soil Particle-Size Fractions after 32 Years of Chemical and Manure Application in a Continuous Maize Cropping System

**DOI:** 10.1371/journal.pone.0152521

**Published:** 2016-03-31

**Authors:** Andong Cai, Hu Xu, Xingfang Shao, Ping Zhu, Wenju Zhang, Minggang Xu, Daniel V. Murphy

**Affiliations:** 1 National Engineering Laboratory for Improving Quality of Arable Land, Institute of Agricultural Resources and Regional Planning, Chinese Academy of Agricultural Sciences, Beijing, 100081, China; 2 College of Agriculture, Guizhou Universities, Guiyang 550025, China; 3 Jilin Academy of Agricultural Sciences, Gongzhuling 136100, China; 4 Soil Biology and Molecular Ecology Group, School of Geography and Environmental Sciences, Institute of Agriculture, University of Western Australia, Crawley, WA 6009, Australia; Chinese Academy of Sciences, CHINA

## Abstract

Long-term manure application is recognized as an efficient management practice to enhance soil organic carbon (SOC) accumulation and nitrogen (N) mineralization capacity. A field study was established in 1979 to understand the impact of long-term manure and/or chemical fertilizer application on soil fertility in a continuous maize cropping system. Soil samples were collected from field plots in 2012 from 9 fertilization treatments (M_0_CK, M_0_N, M_0_NPK, M_30_CK, M_30_N, M_30_NPK, M_60_CK, M_60_N, and M_60_NPK) where M_0_, M_30_, and M_60_ refer to manure applied at rates of 0, 30, and 60 t ha^−1^ yr^−1^, respectively; CK indicates no fertilizer; N and NPK refer to chemical fertilizer in the forms of either N or N plus phosphorus (P) and potassium (K). Soils were separated into three particle-size fractions (2000–250, 250–53, and <53 μm) by dry- and wet-sieving. A laboratory incubation study of these separated particle-size fractions was used to evaluate the effect of long-term manure, in combination with/without chemical fertilization application, on the accumulation and mineralization of SOC and total N in each fraction. Results showed that long-term manure application significantly increased SOC and total N content and enhanced C and N mineralization in the three particle-size fractions. The content of SOC and total N followed the order 2000–250 μm > 250–53μm > 53 μm fraction, whereas the amount of C and N mineralization followed the reverse order. In the <53 μm fraction, the M_60_NPK treatment significantly increased the amount of C and N mineralized (7.0 and 10.1 times, respectively) compared to the M_0_CK treatment. Long-term manure application, especially when combined with chemical fertilizers, resulted in increased soil microbial biomass C and N, and a decreased microbial metabolic quotient. Consequently, long-term manure fertilization was beneficial to both soil C and N turnover and microbial activity, and had significant effect on the microbial metabolic quotient.

## Introduction

Soil organic carbon (SOC) and nitrogen (N) mineralization are fundamental biogeochemical processes that underpin soil fertility and crop production. A better understanding of the mineralization of SOC and N in soils is necessary to improve management of soil fertility, thereby contributing to food security and climate change mitigation. Application of organic amendments, with/without chemical fertilizers [including N, phosphorus (P), and potassium (K)], is recommended as a proven practice to enhance both the accumulation and mineralization capacity of soil organic matter in agricultural systems [1–3]. Manure application can improve soil structure [4] and increase the amount and availability of nutrients for microbial processing [5]. A number of studies have shown that long-term application of organic manure can significantly increase SOC and total N both in bulk soil and particle-size fractions [6, 7]; especially when applied in combination with chemical fertilizers.

The mineralization of SOC and total N in different particle-size fractions is largely regulated by soil organic matter quality (e.g., lignin content and C/N ratio) and the stability which is largely determined by soil inherent properties (e.g., texture and mineralogy) [8]. For example, sands exhibit weak bonding affinities to organic molecules, while organic molecules associated with clay particles (e.g. sesquioxides, layer silicates) can be absorbed by strong ligand exchange and polyvalent action bridges over large surface areas and at numerous reactive positions [9]. Slower SOC turnover times in the clay fraction may be explained by the combined action of an increase in spatial inaccessibility (e.g., due to micro-aggregation) that restricts microbial decomposition, the adsorption of SOC on mineral surfaces, and the consequent chemical change in SOM quality [10–12]. Consequently, SOC within the sand fraction is allocated to the active pool, whereas SOC in silt and clay fractions are defined as the intermediate and passive pools. Various ^13^C natural abundance studies have revealed turnover times of about 15–50 years for SOC stored in the >250 μm fraction and 100–300 years for SOM in the <250 μm fraction [13, 14].

Field management practices can impact on SOC stabilization process through altered aeration associated with mechanical soil disturbance and increased nutrient availability from fertilizer application [1, 15]. A number of studies have shown that long-term application of organic manure significantly increased SOC and total N not only in bulk soil, but also in soil particle-size fractions [6, 7]; especially when manure was applied in combination with chemical fertilizers. Long-term compost application significantly increased the stability of SOC in the 53–250 μm and <53 μm fractions, but not in the >250 μm fraction [16]. It is also reported that the mineralization of SOC and N within particle-size fractions varies with land management practices. Hernández-Hernández and López-Hernández [17] showed that more N was mineralized in the >250 μm fraction than in the <250 μm fraction in cultivated soils, whereas SOC mineralization was significantly higher in the >250 μm fraction than in the <250 μm fraction of the non-cultivated soil. Although, Elliott [18] and Gupta and Germida [19] reported that more SOC and N was mineralized in the >250 μm fraction than in the <250 μm fraction in both cultivated and non-cultivated soils. Hence, there is little available information on the relative contribution of SOC and N mineralization from different particle-size fractions to bulk soil under long-term chemical fertilizer application compared with manure. The objective of this study were to (1) quantify the impact of long-term (32 years) manure application (with/without chemical fertilizer) on the accumulation and distribution of SOC and total N in different particle-size fractions, (2) explore the responses of SOC and N mineralization to manure application in different particle-size fractions, and (3) evaluate the impact of long-term manure application on soil microbial biomass and activity in various soil particle sizes. We focused the study on Mollisols as this soil type plays an important role in grain production in China and covers 75% of the total area (5.9 × 10^6^ ha) cultivated for agricultural production. The overall target was to improve the understanding of manure application on soil fertility improvement within this agro-ecosystem.

## Materials and Methods

### Site description and soil sampling

A long-term agricultural site was established in 1979 by Jilin Academy of Agricultural Sciences. The site is located in Gongzhuling (148°57′E, 42°57′N), Jilin province, Northeast China. The climate is classified as temperate semi-humid monsoon, with mean annual air temperature of 4.5°C and annual precipitation from 450 to 600 mm, with about 70% of precipitation occurring between June and August. This site has 125 to 140 frost-free days and an average 2500 to 2700 h of sunshine each year. Prior to the establishment of the long-term experiment, the field had been intensively cultivated for more than 50 years. The initial (in 1979) topsoil (0–20 cm) had a SOC of 16.1 g kg^-1^, total N of 1.9 g kg^-1^, total P of 1.39 g kg^-1^, and total K of 22.1g kg^-1^. Available N (alkali-hydrolizable), P (Olsen-P), and K (1 mol L^-1^ NH_4_OAc) were 114, 27, and 190 mg kg^-1^, respectively. Soil pH (1:2.5 w/v, distilled water) was 7.6 and soil clay content (<2 μm) was 29.3%.

The long-term field trial consisted of main plots for manure treatments (M_0_, M_30_ and M_60_) and subplots for chemical fertilizer applications (control, N only or NPK). Nine treatments were chosen for this study: M_0_CK, M_0_N, M_0_NPK, M_30_CK, M_30_N, M_30_NPK, M_60_CK, M_60_N, and M_60_NPK. The CK indicates no fertilizer; M_0_, M_30_, and M_60_ refer to livestock manure applied at 0, 30, and 60 t manure ha^−1^ yr^−1^ on a fresh weight basis, respectively; and NPK represents chemical N (urea), P (multiple superphosphate), and K (potassium sulfate) fertilizer, which were applied at 150, 33, and 62 kg ha^-1^ yr^-1^, respectively. The manure used in this experiment was fresh pig and cattle manure compost. The organic carbon content of manure was 10%-20% and total N content was 0.5%-1%. Water content of manure was around 68.7%. Straw was removed at harvest. Each treatment plot (400 m^2^ in area) was in a randomized block design with no replication and managed as a continuous maize cropping system.

During November 2012, we collected three sub-samples of topsoil (0–20 cm) with each sub-sample consisting of 20–40 cores that were collected using a 5-cm-diam auger, randomly sampled from field plots for each treatment [20]. Crop residues on the soil surface were carefully removed before soil sampling. The fresh bulk soil samples (about 2 kg per plot) were gently broken apart by hand and were passed through a 2 mm sieve. Then, a third of the sieved soil was stored at 4°C for subsequent soil microbial biomass carbon (SMB-C) and nitrogen (SMB-N) measurement. The remaining subsample of soil was air-dried for particle-size fractionation and subsequent SOC and N mineralization assays. Subsamples were also sieved <0.15 mm for SOC and total N content measurement.

### Soil particle-size separation

Soil samples from each field plot were separated into three size fractions (2000–250, 250–53, and <53 μm) by the wet-sieving method [21]. Briefly, about 50 g air-dried bulk soil samples (<2 mm) were wetted slowly up to saturation and kept overnight. Then samples were sieved through 250 μm and 53 μm sieves using automated sieving equipment. The wetted soil samples were then shaken for 30 min under water and the material remaining on each of the sieves was collected; with the <53μm fraction being collected by centrifugation. Each collected fraction was oven-dried at 60°C for 48 hours and weighed for the fraction percentage. A sub-sample of each particle-size fraction was then ground to 0.15 mm for SOC and total N content measurement. The remaining soil (not ground) was kept to measure SOC, N mineralization and soil microbial biomass by incubation. SOC and total N of bulk particle-size soil fractions were analyzed using a C/N/H/S-analyzer (EA3000, Italy).

### Incubation experiment for SOC and N mineralization

For each treatment from the long-term trial, the C and N mineralization of different particle-size fractions and bulk soil were determined from laboratory incubations (in triplicate) using the method reported by Steinweg, et al. [22]. Briefly, a 25 g sample of each particle-size soil fraction was mixed with 25 g field-moist bulk soil to provide a source of microbial inoculums from the same field treatment plot. The soil mixture was then pre-incubated at 25°C for 7 days in a 200 mL jar that contained 5 mL of distilled water that was kept separated to the soil and used to increase humidity. After the pre-incubation period, a 10 g subsample of the soil mixture from each jar was extracted with 50 mL of 2 M KCl to examine the initial ammonium (NH_4_^+^) and nitrate (NO_3_^-^) concentrations, which were quantified using a flow injection analyzer (FIAstar 5000, Sweden). The remaining soils were then incubated at 25°C for 12 days in the closed jars, containing an alkali trap (a 50 mL plastic cup containing 20 mL of 0.25 M NaOH). Each jar was regularly opened and ventilated for 5 minutes. After incubation, the final soil samples were subjected to the same KCl extraction to measure NH_4_^+^ and NO_3_^−^ concentrations. Net N mineralization rates were calculated as the difference between the final and initial extractable inorganic N (NH_4_^+^-N + NO_3_^-^-N) concentrations, after adjustment soil from fresh dry weight. SOC mineralization was calculated as the CO_2_-C absorbed in NaOH, as measured by total oxidizable carbon (Multi N/C 3100, Switzerland). SMB-C of bulk soil and particle-size fractions as well as SMB-N of bulk at the end of incubation were measured using the chloroform fumigation—extraction (K_2_SO_4_) method [23].

### Calculation and statistical analysis

Cumulative CO_2_-C and net N mineralization rates were calculated as the percentage of C or N mineralized compared with the total bulk soil SOC and total N content. The microbial quotient was calculated as the percentage of SMB-C to total SOC, while the metabolic quotient (qCO_2_; i.e., microbial efficiency) was calculated as the rate of CO_2_-C evolved per unit of SMB-C. The change in SOC and total N of bulk soil and particle-size fractions (as well as the percentage change) under different treatments were analyzed using analysis of variance (ANOVA) and LSD test at 5% level of significance. Statistical analysis was conducted using SPSS 11.5. A linear regression was used to determine the relationships between the SOC (total N) storage and potential C (N) mineralization in bulk soil and different particle-size fractions after 32 years of manure fertilization.

## Results

### Change in SOC, total N and soil microbial biomass after 32 years of manure application

**Fig 1:**
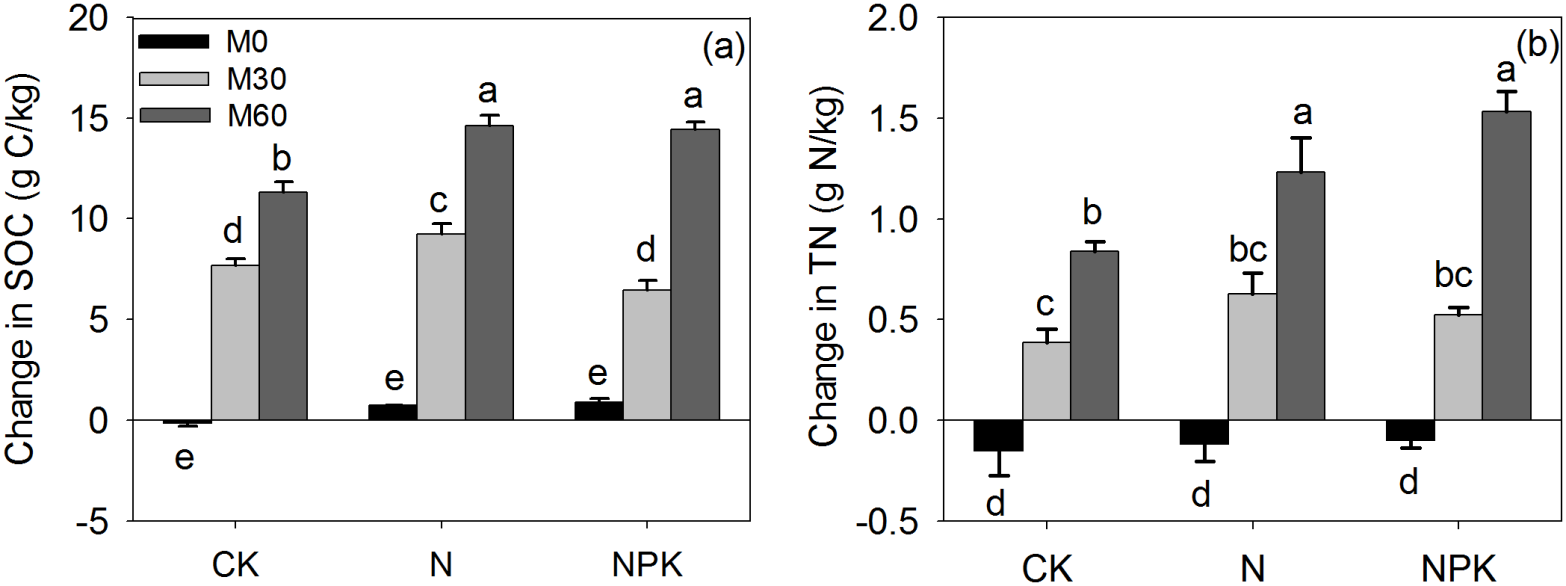
Change in soil organic carbon (SOC, a) and total nitrogen (N) (b) in bulk soil from the control (CK) or soil that has received 32 years of chemical fertilizer application (N, P: phosphorus, K: potassium) and either 0 (M_0_), 30 (M_30_) or 60 (M_60_) t manure ha^-1^ yr^-1^. Note: Different letters above the bars indicate significant differences at 5% probability level. Capped bars are standard error of the mean (n = 3).

The SOC and total N of the control (M_0_CK) remained unchanged over the 32 years of field treatments (16.1 vs. 16.9 g C kg^-1^; 1.9 vs. 1.8 g N kg^-1^; Table 1). Long-term manure application increased SOC by 34–82% and total N by 30–96%, while the application of chemical fertilizer alone (M_0_N and M_0_NPK) had no effect on SOC or total N content, compared with the M_0_CK. The size of the SMB-C increased by 46–196% (and SMB-N increased by 11–91%), in response to 32 years of manure application, whereas there was no effect of chemical fertilizers application alone (Table 1). Nitrogen mineralization significantly increased with increase in manure application rate, while chemical fertilizer or manure application did not influence the C mineralization rate (Table 1). There was no impact of chemical fertilizer application on the C:N of soil microbial biomass (SMB), while increasing manure application resulted in a significant increase in this ratio from a bacterial (M_0_; SMB C:N ratio = 5.6:1) to a fungal (M_60_; SMB C:N ratio = 10.5:1) dominated system (Table 1). There was a tendency for the microbial quotient (qCO_2_) to be higher in the plots that did not received manure; this was significant within the CK and NPK treatments (Table 1). Regardless of chemical fertilizer application, the qCO_2_ was significantly higher (approximately double) in the no manure treatments (M_0_) compared with plots that received manure application. However, there was no significant difference in qCO_2_ between the treatments with M_30_ vs. M_60_. Compared with the initial year soil values, the treatments with M_30_ and M_60_ significantly increased SOC by 7.7 and 11.4 g kg^-1^, and total N by 0.39 and 0.85 g kg^-1^, respectively (Fig 1). This increments represented conversion efficiencies C of 9.16% (M_30_) and 6.80% (M_60_) from manure C to SOC, and of 9.3% (M_30_) and 10.1% (M_60_) from manure-N to soil total N. The effect of combined application of manure and chemical fertilizer was better for SOC and total N sequestration than application manure alone.

**Table 1:** The soil organic carbon (SOC), total nitrogen (N), soil microbial biomass carbon (SMB-C), soil microbial biomass nitrogen (SMB-N), potential C (C_min_) and N (N_min_) mineralization, SMB-C/SMB-N, SMB-C/SOC, and CO_2_/SMB-C (qCO_2_) in bulk soil from the control (CK) or soil that has received 32 years of chemical fertilizer application (N, P: phosphorus, K: potassium) and either 0 (M_0_), 30 (M_30_) or 60 (M_60_) t manure ha^-1^ yr^-1^.

Treatment	SOC	Total N	SMBC	SMBN	C_min_	N_min_	SMBC:SMBN	SMBC:SOC	qCO_2_
	g kg^-1^		mg kg^-1^					%	
CK									
M_0_	16.9e	1.76d	285e	52.1c	151.0a	10.8de	5.5ef	1.7cd	0.54a
M_30_	23.8d	2.29c	611bcd	99.7a	136.1ab	17.3cd	6.1def	2.6a	0.22b
M_60_	27.5b	2.75b	641bc	72.3bc	123.3ab	32.4a	8.9bc	2.3ab	0.19b
N									
M_0_	16.6e	1.79d	349e	55.5bc	155.5a	10.9de	6.3def	2.1abcd	0.45a
M_30_	25.5c	2.54bc	512d	63.7bc	99.2b	14.4cd	8.0cd	2.0bcd	0.19b
M_60_	30.8a	3.16a	687ab	61.8bc	125.6ab	20.2bc	11.1ab	2.2abc	0.18b
NPK									
M_0_	17.0e	1.80d	267 e	53.0c	122.5ab	16.7cd	5.0f	1.6d	0.46a
M_30_	22.7d	2.43bc	544cd	77.2ab	139.6ab	7.0e	7.0de	2.4ab	0.25b
M_60_	30.6a	3.45a	791a	68.6bc	138.6ab	26.1ab	11.5a	2.6a	0.18b

Note: Different letters indicate significant differences at 5% probability level under different treatments.

### SOC and total N in particle-size fractions

**Fig 2:**
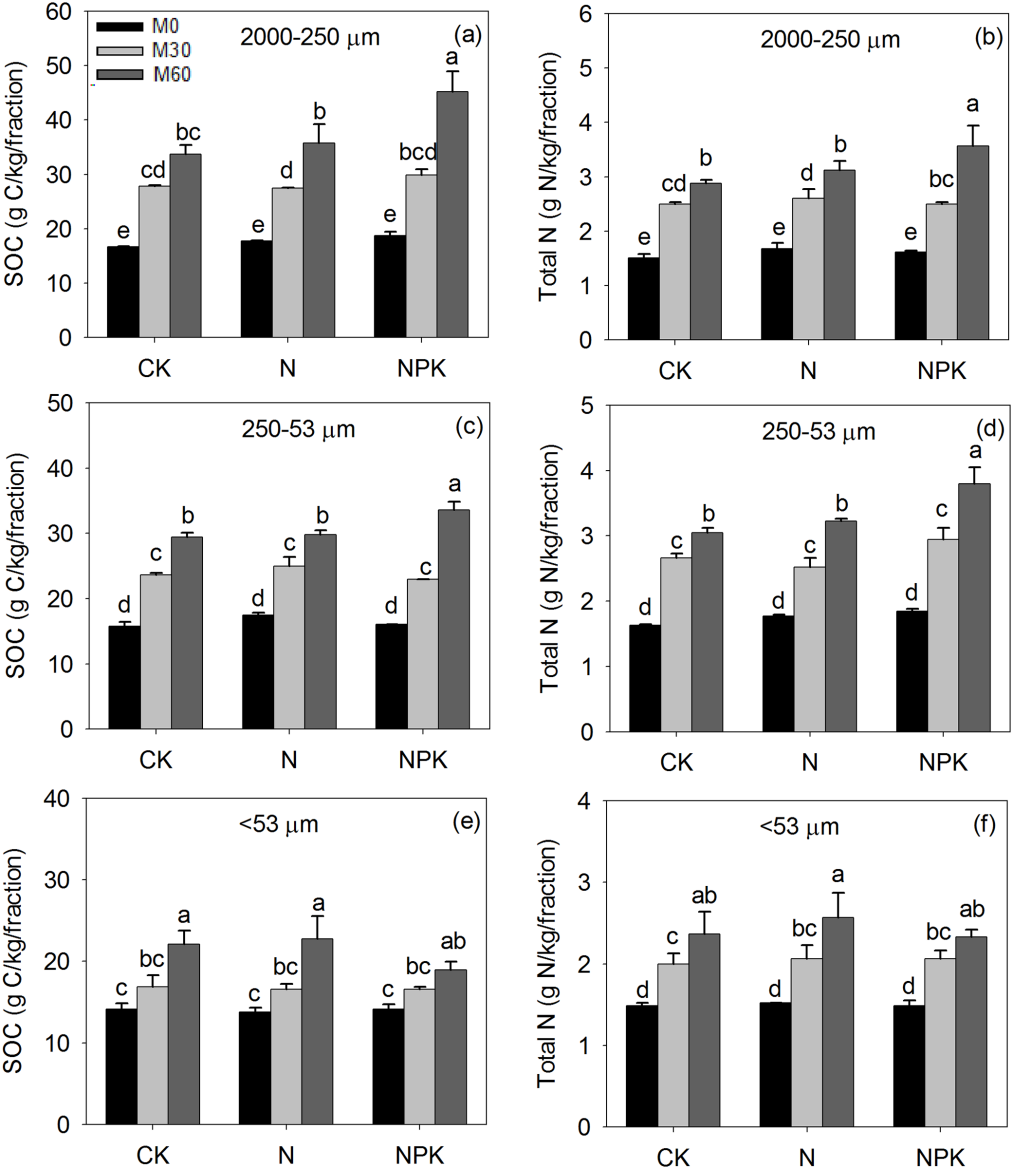
The content of soil organic carbon (SOC) in 2000–250 μm (a), 250–53 μm (c), <53 μm (e) fraction and total nitrogen (N) in 2000–250 μm (b), 250–53 μm (d), <53 μm (f) fraction from the control (CK) or soil that received 32 years of chemical fertilizer application (N, P: phosphorus, K: potassium) and either 0 (M_0_), 30 (M_30_) or 60 (M_60_) t manure ha^-1^ yr^-1^. Note: Different letters above the bars indicate significant differences at 5% probability level. Capped bars are standard error of the mean (n = 3).

**Fig 3:**
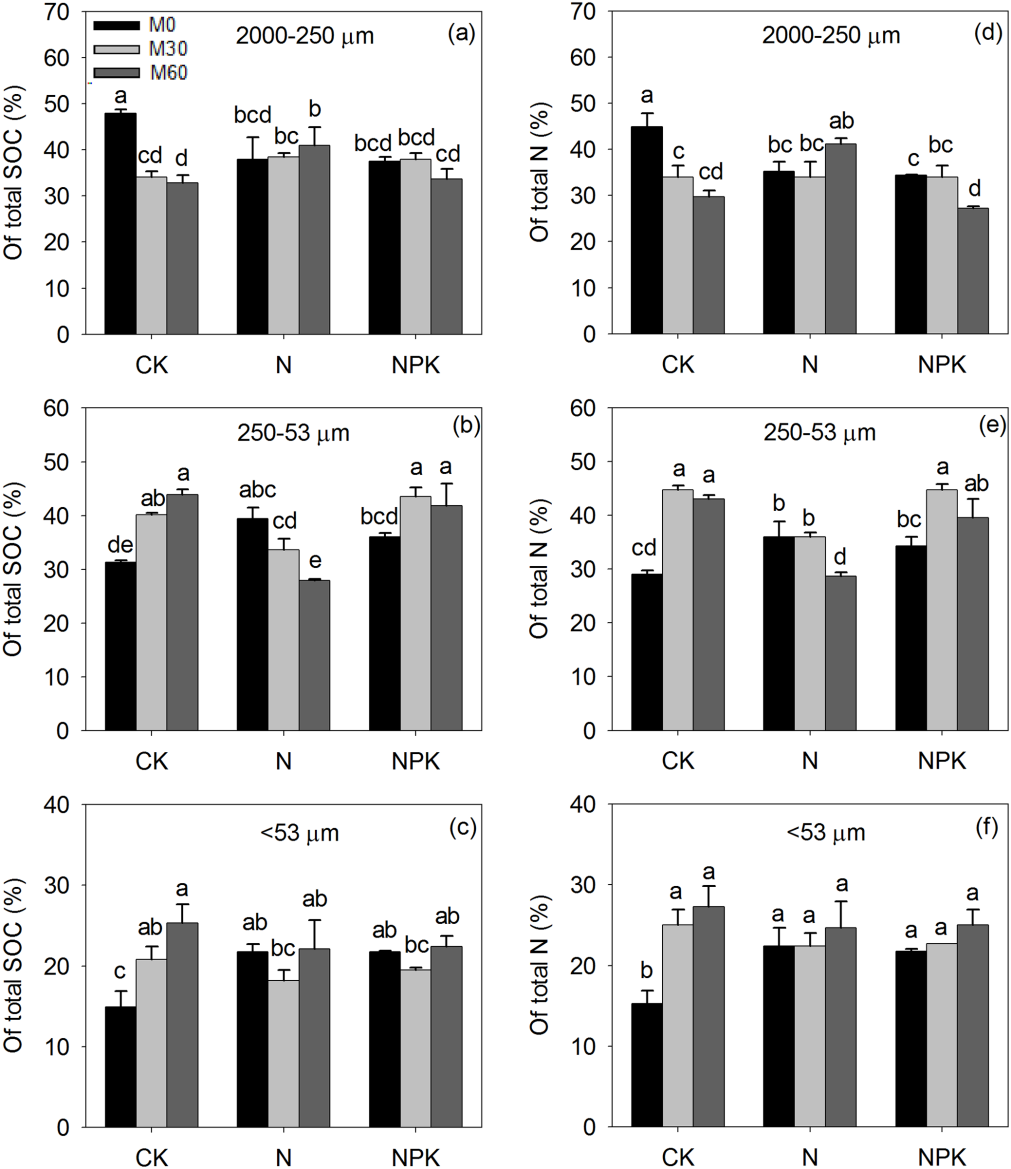
The percent (%) of soil organic carbon (SOC) in 2000–250 μm (a), 250–53 μm (c), <53 μm (e) fraction and total nitrogen (N) in 2000–250 μm (b), 250–53 μm (d), <53 μm (f) fraction from the control (CK) or soil that received 32 years of chemical fertilizer application (N, P: phosphorus, K: potassium) and either 0 (M_0_), 30 (M_30_) or 60 (M_60_) t manure ha^-1^ yr^-1^. Note: Different letters above the bars indicate significant differences at 5% probability level. Capped bars are standard error of the mean (n = 3).

Fractionation recovery rate of total soil weight of the three fractions ranged from 96.1–101.7%; the recovery rate of SOC in the three fractions ranged from 90.2–101.8%, and that of total N ranged from 89.0–103.4%. Long-term manure with/without chemical fertilizer application significantly increased the content of SOC and total N in all soil particle-size fractions, while application of chemical fertilizer on its own (M_0_N, M_0_NPK) did not alter SOC or total N content in any soil particle-size fractions compared with the control (CKM_0_) (Fig 2). Compared with no manure application (M_0_), the 30 and 60 t ha^-1^ annual manure application rates increased the SOC in soil fractions by 20–60% (M_30_) and 52–115% (M_60_), and increased total N by 34–57% (M_30_) and 61–99% (M_60_). The greatest amount of SOC (45.1 g C kg^-1^ fraction) and total N (4.1 g N kg^-1^ fraction) occurred in the 2000–250 μm particle-size fraction under the M_60_NPK treatment. There was no significantly difference in the soil C:N ratio of the 2000–250 μm fraction among treatments (C:N = 10.5:1), while manure combined with NPK application decreased the soil C:N ratio of the 250–53 μm (C:N = 9.8:1) and <53 μm fractions to some extent (C:N = 8.8:1). The 2000–250 μm and 250–53 μm particle-size fractions contained approximately 37% of the total SOC and total N pools among the three particle-size fractions, regardless of the difference in management treatments (Fig 3).

**Fig 4:**
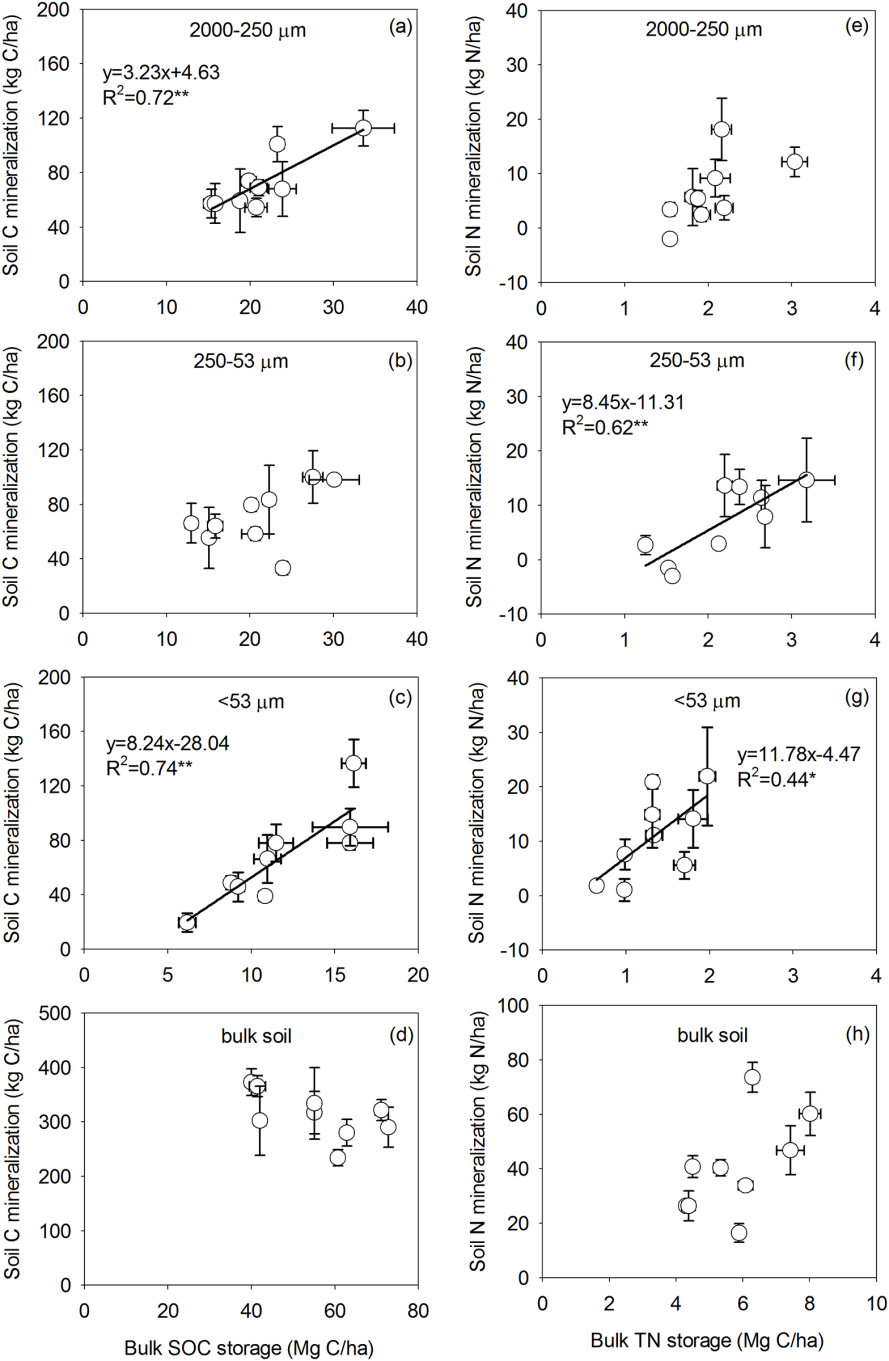
Relationship between soil organic carbon (SOC) storage vs potential carbon (C) mineralization, and total N storage, potential nitrogen (N) mineralization in 2000–250 μm, 250–53 μm, <53 μm fraction and bulk soil after 32 years of manure fertilization.

The amount of SOC and N mineralized differed among particle-size fractions and under different fertilizer application treatments (Table 2). For the CK treatments, there were no differences among the three soil fractions under manure application. However, there was more C mineralized from the <53 μm fraction with manure applied at 60 t ha^-1^ yr^-1^ (M_60_), compared with no manure (M_0_, Table 2). As a general trend N mineralization increased with manure application, showing more N mineralized from <53 μm fraction under the M_30_NPK and M_60_NPK treatments. There was no relationship between the storage of SOC or total N and the C or N mineralization from the bulk soil (Fig 4). Interestingly, N mineralization was strongly related to total N storage in the 250–53 μm and <53 μm fractions and C mineralization was significantly positive related to SOC storage in the 2000–250 μm and <53 μm fractions.

**Table 2:** The potential pool of soil organic carbon (SOC) and nitrogen (N) mineralized in different particle-size fractions from the control (CK) or soil that received 32 years of chemical fertilizer application (N, P: phosphorus, K: potassium) and either 0 (M_0_), 30(M_30_) or 60 (M_60_) t manure ha^-1^ yr^-1^ (mg kg^-1^ soil).

Treatment	SOC mineralization			Soil N mineralization		
	2000–250 μm	250–53 μm	<53 μm	2000–250 μm	250–53 μm	<53 μm
CK						
M_0_	30.5bc	27.1abcd	8.5e	1.1bc	1.2abc	0.9cd
M_30_	25.9bc	36.3abc	34.0bc	2.5bc	5.7a	6.6abc
M_60_	24.1c	43.9a	34.6bc	2.4bc	3.5abc	2.5cd
N						
M_0_	23.9c	26.6abcd	20.6cde	1.5bc	- 1.2c	3.1bcd
M_30_	42.2ab	24.5bcd	28.3bcd	7.7a	5.8a	4.7abcd
M_60_	48.6a	34.1abc	38.7b	5.2ab	1.2abc	6.2abcd
NPK						
M_0_	23.1c	22.3cd	19.0de	- 0.7c	- 0.6bc	0.5d
M_30_	29.1bc	13.6d	16.7de	3.9ab	4.7ab	8.7ab
M_60_	29.6bc	42.3ab	59.1a	1.7bc	6.3a	9.5a
Average ^a^	33.3	32.5	35.2	3.9	4.5	6.4

Note: Different letters indicate significant differences at 5% probability level within the same particle-size fraction.

^a^ the average represent manure treatments.

Long-term application of manure significantly increased SMB-C in all soil fractions with without manure. However, chemical fertilizer alone application showed no effect on SMB-C (Table 3). Compared with long-term application of chemical fertilizer, manure application had significant decreased the microbial metabolic quotient (Table 1). The phenomenon of microbial metabolic quotient decreasing mainly occurred in the 250–53 μm and <53 μm fractions (Table 3).

**Table 3:** Soil microbial biomass carbon(SMB-C) and CO_2_/SMB-C (qCO_2_) in different particle-size fractions from the control (CK) or soil that received 32 years of chemical fertilizer application (N: nitrogen, P: phosphorus, K: potassium) and either 0 (M_0_), 30(M_30_) and 60 (M_60_) t manure ha^-1^ yr^-1^.

Treatment	SMB-C (mg kg^-1^ soil)			CO_2_/SMB-C		
	2000–250 μm	250–53 μm	<53 μm	2000–250 μm	250–53 μm	<53 μm
CK						
M_0_	68cd	32de	18e	0.46b	0.84b	0.49c
M_30_	64cd	56cd	55bc	0.38b	0.64bc	0.61bc
M_60_	79bc	99ab	81b	0.31b	0.45cd	0.43c
N						
M_0_	31e	22e	25de	1.11a	1.34a	1.06ab
M_30_	104ab	82bc	63b	0.41b	0.31cd	0.51c
M_60_	108a	62cd	34cde	0.46b	0.55bc	1.15a
NPK						
M_0_	45de	42de	31cde	0.50b	0.50bcd	0.69abc
M_30_	84abc	78bc	53bcd	0.36b	0.18d	0.33c
M_60_	68cd	125a	126a	0.47b	0.37cd	0.51c

Note: Different letters indicate significant differences at 5% probability level within the same particle-size fraction.

## Discussion

### The effect of manure application on SOC and total N distribution

Long-term manure application, with/without chemical fertilizer application, significantly increased SOC and total N in bulk soil and all particle-size fractions, indicating that manure application was effective at building new soil organic matter in this agro-ecosystem [24, 25]. The SOC and total N content in the 2000–250 μm and <53 μm fractions (and thus bulk soil) was further increased when manure was applied in combination with chemical nutrition of N, P, and K (M_60_NPK) compared to manure alone (M_60_CK). Therefore, for soil fertility, the inorganic nutrient status played an important role in the extent of soil C sequestration. Our findings were consistent with findings from Zhu, et al. [26], who showed that while mineral fertilizer application can maintain high yields, a combination of chemical fertilizers plus manure was required to enhance soil C sequestration. The content of SOC and total N in soil particle-size fractions declined with a decrease in particle-size, indicating that larger-size soil fractions were the main pool of SOC and total N [27], with the less decomposable SOC and N associated with fine soil particles [28].

## The effect of manure application on SOC and N mineralization

The amount of SOC and N mineralization differed according to the size of soil particle-size fractions. Our study revealed that the amount of SOC and N mineralization followed the order: 53 μm> 250–53 μm> 2000–250 μm fraction. However, our findings contrasted with findings that reported organic C in soil aggregates to be more stable in small particle-size fraction than in larger particle-size fraction [29]. A similar amount of C was mineralized from the soil particle-size fractions. A number of studies have shown that SOC stability in aggregates generally increases with decreasing aggregate size [30, 31], primarily due to physical protection in micro aggregates and/or physico-chemical stabilization in the silt + clay fraction [32, 33]. Therefore, soil C sequestration was variable due to physical protection in the <250 μm fraction or physico-chemical stabilization in the <53 μm fraction, depending on both soil types and management measures [34, 35]. The N mineralized in the <53 μm fraction was about 1.5 times greater than that in the other two fractions, suggesting that smaller particle-size fractions contain a larger proportion of readily mineralizable organic N than larger particle-size fraction in Mollisols. The significant linear relationships between SOC storage and C mineralization (and similarly total N versus N mineralization) in the <53 μm particle-size fraction indicated that this fraction had the greatest capacity for SOC and N mineralization.

### The effect of manure application on soil microbial biomass and qCO_2_

Our findings clearly indicated that the addition of manure increased SMB-C within all fractions and bulk soil. However, the highest level of SMB-C occurred in treatment plots that received the highest manure level (M_60_) in combination with chemical fertilizers (M_60_N; M_60_NPK), indicating a nutrient limitation to the size of the microbial biomass in other treatments. We measured more SMB-C in the larger particle-size fractions, which supported that microbial biomass had different distributions in different particle-size fractions [36]. Similarly, Franzluebbers and Arshad [37] have also reported higher SMB-C in the>250 μm fraction than in the <250 μm fraction, probably due to differences in microbial accessibility to substrates resulting from fraction stability. On the contrary, other researcher has reported same or higher microbial activity in the <250 μm fraction [38]. The average ratio of SMB-C to SMB-N of fungi is about 15:1, versus 6:1 for bacteria. We found 32 years of manure application caused a shift from a bacterial to a fungal dominated population, which promoted the transformation of soil organic C and N storage [39, 40].

Long-term manure and chemical fertilizer application had a significant influence on the SMB-C and SOC mineralization. Manure application stimulates soil microbial activity due to the increased annual additions of fresh C inputs [41]. The qCO_2_ provides a measure of the specific metabolic activity, which varies according to the composition and physiological state of the microbial community, the availability of substrates, and various abiotic factors. Our finding that reduction by the addition of manure compared with without manure in qCO_2_ indicated lower catabolic demand of the soil microbial community [42]. It is believed that high soil qCO_2_ might be attributed to lower availability of soil nutrients[43], whereas the lower qCO_2_ under organic practices might be due to the protective capacity of the microbial biomass [44]. Either way, results suggested a more stable environment for microbial activity when manure was applied to soil. Our findings illustrate that long-term manure application enhanced SOC and total N stabilization, especially when it was applied in combination with chemical fertilizers to enable improved microbial efficiency.

The conversion efficiency from manure C into SOC in Mollisols (9.4±3.1% for M_30_ and 8.1 ± 2.7% for M_60_) was at the range of global manure C retention coefficient of 12±4% [45]. But it was much lower than the result from similar temperate climate with high clay content (23±15%) [46, 47]. The similar soil organic carbon sequestration efficiency for both the 30 and 60 t manure ha^-1^ yr^-1^ application rates used in our study indicate that this soil has not yet reached its soil C saturation capacity and thus can sequester more CO_2_ as SOC.

## Supporting Information

S1 Dataset S1 Dataset was the data of SOC, total N, potential C and N mineralization, soil microbial biomass carbon, nitrogen and metabolic quotient. This data contained two parts: (1). Soil organic carbon, total nitrogen, potential C and N mineralization in bulk soil and different particle-size fractions from the control (CK) or soil that received 32 years of chemical fertilizer application (N: nitrogen, P: phosphorus, K: potassium) and either 0 (M0), 30 (M30) or 60 (M60) t manure ha^-1^ yr^-1^. (2). Soil microbial biomass carbon and microbial metabolic quotient in bulk soil and different particle-size fractions from the control (CK) or soil that received 32 years of chemical fertilizer application (N: nitrogen, P: phosphorus, K: potassium) and either 0 (M0), 30(M30) and 60 (M60) t manure ha^-1^ yr^-1^.
(XLSX)

## References

[1] Aoyama M, Angers DA, N'Dayegamiye A (1999) Particulate and mineral-associated organic matter in water-stable aggregates as affected by mineral fertilizer and manure applications. Can J Soil Sci 79: 295–302.

[2] Liang B, Yang X, He X, Murphy DV, Zhou J (2012) Long-term combined application of manure and NPK fertilizers influenced nitrogen retention and stabilization of organic C in Loess soil. Plant Soil 353: 249–260.

[3] Jiang G, Xu M, He X, Zhang W, Huang S, Yang X, et al (2014) Soil organic carbon sequestration in upland soils of northern China under variable fertilizer management and climate change scenarios. Global Biogeochem Cy 28: 319–333.

[4] Haynes RJ, Naidu R (1998) Influence of lime, fertilizer and manure applications on soil organic matter content and soil physical conditions: a review. Nutr Cycl Agroecosys 51: 123–137.

[5] Mikha MM, Rice CW (2004) Tillage and manure effects on soil and aggregate-associated carbon and nitrogen. Soil Sci Soc Am J 68: 809–816.

[6] Huang S, Peng X, Huang Q, Zhang W (2010) Soil aggregation and organic carbon fractions affected by long-term fertilization in a red soil of subtropical China. Geoderma 154: 364–369.

[7] Meng Q, Sun Y, Zhao J, Zhou L, Ma X, Zhou M, et al (2014) Distribution of carbon and nitrogen in water-stable aggregates and soil stability under long-term manure application in solonetzic soils of the Songnen plain, northeast China. J Soil Sediment 14: 1041–1049.

[8] Parfitt RL, Salt GJ (2001) Carbon and nitrogen mineralisation in sand, silt, and clay fractions of soils under maize and pasture. Soil Res 39: 361–371.

[9] Sposito G, Skipper NT, Sutton R, Park S-h, Soper AK, Greathouse JA (1999) Surface geochemistry of the clay minerals. P Natl A Sci 96: 3358–3364. 10.1073/pnas.96.7.3358 PMC34275 10097044

[10] Hassink J, Bouwman LA, Zwart KB, Bloem J, Brussaard L (1993) Relationships between soil texture, physical protection of organic matter, soil biota, and c and n mineralization in grassland soils. Geoderma 57: 105–128.

[11] Wattel‐Koekkoek E, Buurman P, Van Der Plicht J, Wattel E, Van Breemen N (2003) Mean residence time of soil organic matter associated with kaolinite and smectite. Eur J Soil Sci 54: 269–278.

[12] Kleber M, Mertz C, Zikeli S, Knicker H, Jahn R (2004) Changes in surface reactivity and organic matter composition of clay subfractions with duration of fertilizer deprivation. Eur J Soil Sci 55: 381–391.

[13] Besnard E, Chenu C, Balesdent J, Puget P, Arrouays D (1996) Fate of particulate organic matter in soil aggregates during cultivation. Eur J Soil Sci 47: 495–503.

[14] John B, Yamashita T, Ludwig B, Flessa H (2005) Storage of organic carbon in aggregate and density fractions of silty soils under different types of land use. Geoderma 128: 63–79.

[15] Pagliai M, Vignozzi N, Pellegrini S (2004) Soil structure and the effect of management practices. Soil Till Res 79: 131–143.

[16] Yu H, Ding W, Luo J, Geng R, Ghani A, Cai Z (2012) Effects of long-term compost and fertilizer application on stability of aggregate-associated organic carbon in an intensively cultivated sandy loam soil. Biol Fertil Soils 48: 325–336.

[17] Hernández-Hernández RM, López-Hernández D (2002) Microbial biomass, mineral nitrogen and carbon content in savanna soil aggregates under conventional and no-tillage. Soil Biol Biochem 34: 1563–1570.

[18] Elliott ET (1986) Aggregate Structure and Carbon, Nitrogen, and Phosphorus in Native and Cultivated Soils1. Soil Sci Soc Am J 50: 627–633.

[19] Gupta VVSR, Germida JJ (1988) Distribution of microbial biomass and its activity in different soil aggregate size classes as affected by cultivation. Soil Biol Biochem 20: 777–786.

[20] Chen Y, Zhang X, He H, Xie H, Yan Y, Zhu P, et al (2009) Carbon and nitrogen pools in different aggregates of a Chinese Mollisol as influenced by long-term fertilization. J Soil and Sediment 10: 1018–1026.

[21] Cambardella CA, Elliott ET (1992) Particulate soil organic matter changes across a grassland cultivation sequence. Soil Sci Soc Am J 56: 777–783.

[22] Steinweg JM, Fisk M, McAlexander B, Groffman P, Hardy J (2008) Experimental snowpack reduction alters organic matter and net N mineralization potential of soil macroaggregates in a northern hardwood forest. Biol Fertil Soils 45: 1–10.

[23] Wu J, Lin Q, Huang Q, Xiao H (2006) Soil microbial biomass-methods and application. China Meteorological Press (in Chinese), Beijing 12: 54–78.

[24] Triberti L, Nastri A, Giordani G, Comellini F, Baldoni G, et al (2008) Can mineral and organic fertilization help sequestrate carbon dioxide in cropland? Eur J Agron 29: 13–20.

[25] Whalen JK, Chang C (2002) Macroaggregate characteristics in cultivated soils after 25 annual manure applications. Soil Sci Soc Am J 66: 1637–1647.

[26] Zhu P, Ren J, Wang L, Zhang X, Yang X, MacTavish D (2007) Long-term fertilization impacts on corn yields and soil organic matter on a clay-loam soil in Northeast China. J Plant Nutr Soil Sci 170: 219–223.

[27] Cambardella CA, Elliott ET (1994) Carbon and nitrogen dynamics of soil organic matter fractions from cultivated grassland soils. Soil Sci Soc Am J 58: 123–130.

[28] Puget P, Chenu C, Balesdent J (1995) Total and young organic matter distributions in aggregates of silty cultivated soils. Eur J Soil Sci 46: 449–459.

[29] Allison SD, Jastrow JD (2006) Activities of extracellular enzymes in physically isolated fractions of restored grassland soils. Soil Biol Biochem 38: 3245–3256.

[30] Ashman MR, Hallett PD, Brookes PC (2003) Are the links between soil aggregate size class, soil organic matter and respiration rate artefacts of the fractionation procedure? Soil Biol Biochem 35: 435–444.

[31] Puget P, Chenu C, Balesdent J (2000) Dynamics of soil organic matter associated with particle-size fractions of water-stable aggregates. Eur J Soil Sci 51: 595–605.

[32] Six J, Elliott ET, Paustian K (2000) Soil macroaggregate turnover and microaggregate formation: a mechanism for C sequestration under no-tillage agriculture. Soil Biol Biochem 32: 2099–2103.

[33] Du Z-l, Wu W-l, Zhang Q-z, Guo Y-b, Meng F-q (2014) Long-term manure amendments enhance soil aggregation and carbon saturation of stable pools in North China plain. J Integr Agriculture 13: 2276–2285.

[34] Hassink J (1997) The capacity of soils to preserve organic C and N by their association with clay and silt particles. Plant Soil 191: 77–87.

[35] Six J, Conant RT, Paul EA, Paustian K (2002) Stabilization mechanisms of soil organic matter: Implications for C-saturation of soils. Plant Soil 241: 155–176.

[36] Seech AG, Beauchamp EG (1988) Denitrification in soil aggregates of different sizes. Soil Sci Soc Am J 52: 1616–1621.

[37] Franzluebbers AJ, Arshad MA (1997) Soil microbial biomass and mineralizable carbon of water-stable aggregates. Soil Sci Soc Am J 61: 1090–1097.

[38] Mendes IC, Bandick AK, Dick RP, Bottomley PJ (1999) Microbial biomass and activities in soil aggregates affected by winter cover crops. Soil Sci Soc Am J 63: 873–881.

[39] Suzuki C, Nagaoka K, Shimada A, Takenaka M (2009) Bacterial communities are more dependent on soil type than fertilizer type, but the reverse is true for fungal communities. Soil Sci Plant Nutr 55: 80–90.

[40] Kamaa M, Mburu H, Blanchart E, Chibole L, Chotte J-L, Kibunja C, et al (2011) Effects of organic and inorganic fertilization on soil bacterial and fungal microbial diversity in the Kabete long-term trial, Kenya. Biol Fert Soils 47: 315–321.

[41] Liu E, Yan C, Mei X, He W, Bing SH, Ding L, et al (2010) Long-term effect of chemical fertilizer, straw, and manure on soil chemical and biological properties in northwest China. Geoderma 158: 173–180.

[42] Ding X, Han X, Liang Y, Qiao Y, Li L, Li N (2012) Changes in soil organic carbon pools after 10 years of continuous manuring combined with chemical fertilizer in a Mollisol in China. Soil Till Res 122: 36–41.

[43] Agnelli A, Ugolini FC, Corti G, Pietramellara G (2001) Microbial biomass-C and basal respiration of fine earth and highly altered rock fragments of two forest soils. Soil Biol Biochem 33: 613–620.

[44] Pascual JA, García C, Hernandez T, Ayuso M (1997) Changes in the microbial activity of an arid soil amended with urban organic wastes. Biol Fertil Soils 24: 429–434.

[45] Maillard E, Angers DA (2014) Animal manure application and soil organic carbon stocks: a meta-analysis. Global Change Biol 20: 666–679. 10.1111/gcb.12438 24132954

[46] Collins HP, Rasmussen PE, Douglas CL (1992) Crop Rotation and Residue Management Effects on Soil Carbon and Microbial Dynamics. Soil Sci Soc Am J 56: 783–788.

[47] Johnson JMF, Franzluebbers AJ, Weyers SL, Reicosky DC (2007) Agricultural opportunities to mitigate greenhouse gas emissions. Environ Pollut 150: 107–124. 17706849 10.1016/j.envpol.2007.06.030

